# Outcome prediction in disorders of consciousness: the role of coma recovery scale revised

**DOI:** 10.1186/s12883-019-1293-7

**Published:** 2019-04-18

**Authors:** Lucia Francesca Lucca, Danilo Lofaro, Loris Pignolo, Elio Leto, Maria Ursino, Maria Daniela Cortese, Domenico Conforti, Paolo Tonin, Antonio Cerasa

**Affiliations:** 1 S. Anna Institute and Research in Advanced Neurorehabilitation (RAN), 88900 Crotone, Italy; 2 Dipartimento di Ingegneria Meccanica, Energetica e Gestionale – DIMEG, UNICAL, Arcavata di Rende (CS), Rende, Italy; 3 Kidney and Transplantation Research Center, Annunziata Hospital, Cosenza, Italy; 4 Neuroimaging Unit, IBFM-CNR, 88100 Catanzaro, Italy

## Abstract

**Background:**

To evaluate the utility of the revised coma remission scale (CRS-r), together with other clinical variables, in predicting emergence from disorders of consciousness (DoC) during intensive rehabilitation care.

**Methods:**

Data were retrospectively extracted from the medical records of patients enrolled in a specialized intensive rehabilitation unit. 123 patients in a vegetative state (VS) and 57 in a minimally conscious state (MCS) were included and followed for a period of 8 weeks. Demographical and clinical factors were used as outcome measures. Univariate and multivariate Cox regression models were employed for examining potential predictors for clinical outcome along the time.

**Results:**

VS and MCS groups were matched for demographical and clinical variables (i.e., age, aetiology, tracheostomy and route of feeding). Within 2 months after admission in intensive neurorehabilitation unit, 3.9% were dead, 35.5% had a full recovery of consciousness and 66.7% remained in VS or MCS. Multivariate analysis demonstrated that the best predictor of functional improvement was the CRS-r scores. In particular, patients with values greater than 12 at admission were those with a favourable likelihood of emergence from DoC.

**Conclusions:**

Our study highlights the role of the CRS-r scores for predicting a short-term favorable outcome.

## Background

After a period of coma resulting from severe acquired cerebrovascular injury of vascular, traumatic or anoxic origin, patients may present an evolution through three ascending disorders of consciousness (DoC) levels: coma, vegetative state (VS) and minimal conscious state (MCS) [1]. The vegetative state, also known as “unresponsive wakefulness syndrome” [2], is a clinical condition of wakefulness without awareness, in which eyes are open but there is no evidence of consciousness as manifested by volitional responses. The MCS is a condition of severely altered consciousness in which minimal but definite behavioural evidence of environmental awareness or himself is demonstrated [3]. The MCS is usually considered as a transitional state characterized by an improvement of consciousness where patients showed more than automatic behavior or purely reflex as observed in VS. Unfortunately, some patients in MCS may progress slowly while others remain in this condition even years or decades. Otherwise, emergence from MCS occurs when the patient is able to reliably communicate through verbal or gestural yes–no responses, or is able to demonstrate the use of two or more objects in a functional manner [3].

One of the main targets in the clinical management of patients with DoC is to identify which medical prognostic features might best predict long-term neurologic and functional positive outcome [4]. This was done in order to determine: i) algorithmic approaches to patient treatment; ii) the optimal clinical care and setting to improve outcomes and iii) the risk of long-term severe disability and institutionalization, which increases hospital costs [5].

One of the best diagnostic and prognostic tools useful to distinguish MCS from VS is the Coma Recovery Scale-Revised (CRS-r) [6]. This is a well-known standardized assessment measure designed to detect subtle changes in neurobehavioral status of DoC patients. This comprised of 6 subscales assessing auditory, visual, motor, oromotor, communication, and arousal processes organized in 29 hierarchically items [6]. The reliability of the CRS-r in monitoring conscious awareness evolution has been widely demonstrated. This scale strongly correlated with clinical outcome at discharge and its scores showed excellent concurrent validity with others well-known neurobehavioral scales, such as the Glasgow Outcome Scale [7] and the Disability Rating Scale [8].

There are several longitudinal studies assessing the best prognostic factors of emergence from DoC [9–21]. Generally, three clinical features are strictly correlated with clinical evolution: age, aetiology and degree of severity of neurological impairment. Older age is known to negatively influence outcome both in brief and very long-term longitudinal studies. In patients with traumatic aetiology, increasing age is significantly associated with unfavourable outcome at 6 months, independent of other prognostic factors [6, 7]. Again, traumatic brain-injured (TBI) patients have a better prognosis in terms of survival, recovery of consciousness and function with respect to other aetiologies [15–19]. Finally, MCS patients have been found to have a better prognosis than those in VS [20, 21].

Despite this large amount of evidence, there is a paucity of studies assessing the role of CRS-r in predicting clinical evolution in DoC patients. For this reason, we performed a retrospective observational study in a large cohort of MCS/VS patients with heterogeneous aetiologies.

## Methods

### Subjects

**Fig. 1:**
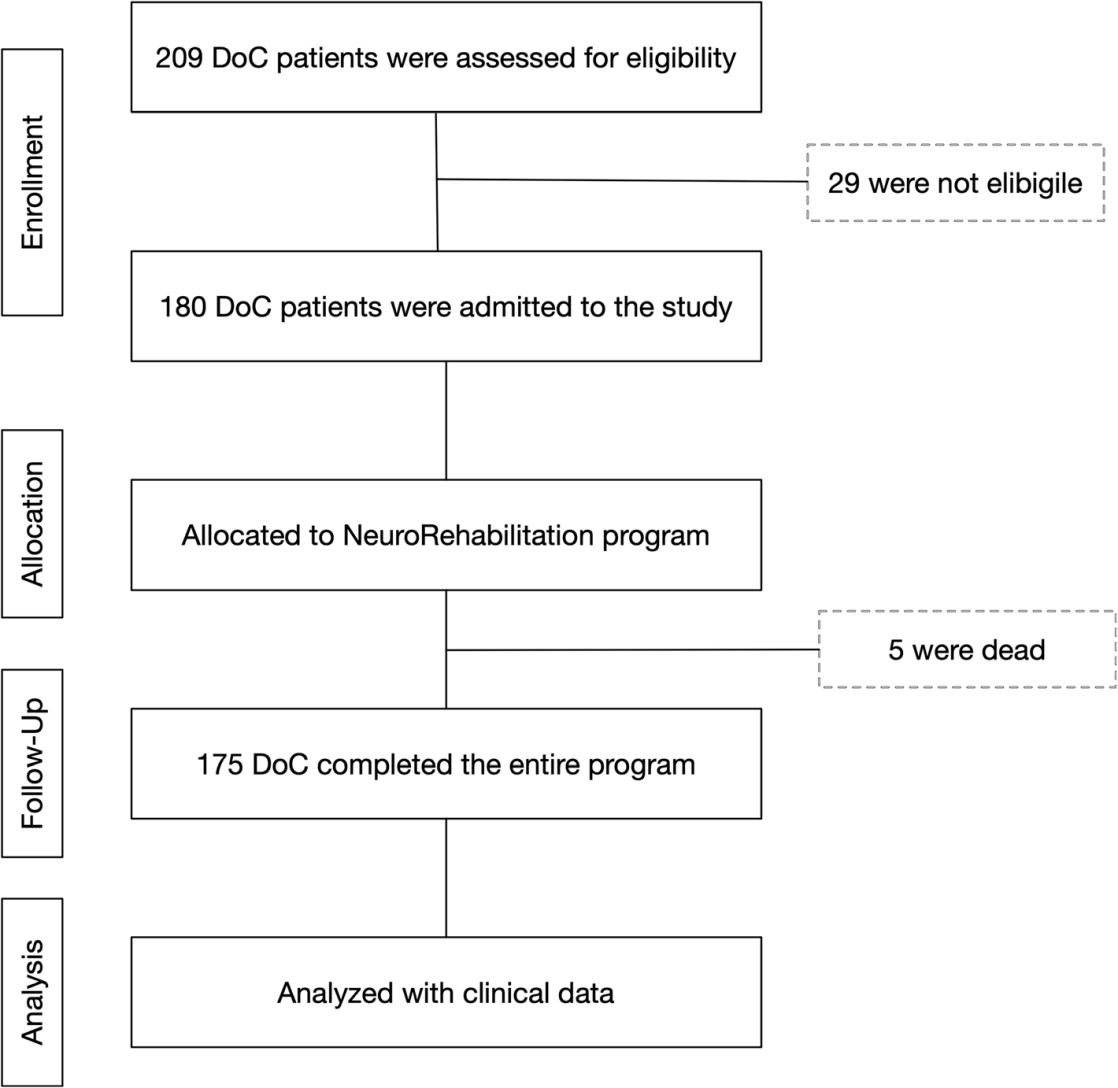
Flow diagram of participant recruitment and participation in the study

We enrolled patients with DoC following acute acquired brain injury of traumatic, vascular, anoxic origin. All patients were consecutively admitted to the intensive rehabilitation unit (IRU) of the Institute S. Anna (Crotone, Italy) between January 2010 and December 2017. All patients were transferred directly from the intensive care unit or neurosurgery, after the medical and neurosurgery complications have been stabilized within a maximum of seven days from the transfer request. From an initial cohort of 209 DoC patients we enrolled only those who fulfilled the following criteria: clinical diagnosis at admission of VS or MCS according to standard diagnostic criteria [3, 22]; (2) acute traumatic, vascular, or anoxic brain injury, identified on the basis of medical records relative to the acute phase and 3) age > 18 years. Exclusion criteria were the presence of a premorbid history of psychiatric or neurodegenerative diseases and patients with mixed aetiology (e.g., both traumatic and anoxic brain injury). From the initial cohort, 180 DoC patients were selected for observational evaluation of the rehabilitation program (Fig. 1).

### Design and procedure

This was a retrospective observational study of patients with DoCs admitted to the IRU from January 2010 to June 2017. Data were extracted retrospectively from electronic patient charts. All patients were tested within 48 h of admission by experienced neurologists and neuropsychologists who were blind to any other result. In order to evaluate the proportion of patients emerged during the follow-up and the factors associated with clinical changes patients underwent clinical evaluation weekly for a period of eight weeks. For investigating possible predictors for the functional and behavioural outcome, several demographical and clinical characteristics were collected: age, sex, aetiology and CRS-r scores, the presence of tracheostomy tube, route of feeding, the time between event and hospitalization in rehabilitation. The study was approved by the Ethical Committee of the University “Magna Graecia” of Catanzaro, according to the Helsinki Declaration.

### Outcome measures

Functional outcome was assessed at admission using the CRS-r. The CRS-r is a behavioural test to quantify levels of consciousness and ranges from 0 (deep coma) to 23 (able to follow commands and to use objects purposefully) points comprising six subcategories: alertness and attention, motor response, response to acoustic stimuli, response to visual stimuli, response to tactile stimuli, and verbal response [23]. Patients were included if they had a CRS score corresponding to VS or MCS upon admission. The temporal pattern of CRS improvements was analyzed by determining the week during which the first significant CRS increase occurs.

### Clinical treatment

During the 8-weeks period in the IRU all patients received a program of physical respiratory rehabilitation, passive mobilization, sitting posture conditioning, passive verticalization, training step pattern and speech therapies for at least 2 h/day. Moreover, patients underwent unimodal sensory stimulation to promote specific cognitively mediated responses [24]. The stimulation was applied for eight weeks following a program including all sensorial fields (auditory, visual, tactile, olfactory) [25] Each stimulus was applied alternatively at right and left for six-time (three for each side) and the response of the patients was annotated. The visual and auditory stimulation were applied when the selected parameters of heart rate variability were in a specific range of intervals to have a higher probability of response in DoC patients [26]. Again, the treatment program included management of tone problems, autonomic disturbances and other problems that are common in this population. If necessary due to spasticity or contractures, patients receive therapy with injections of botulinum. Overall, our main interest concerns basic care and secondary medical conditions that can emerge during neurorehabilitation period [17].

### Statistical analysis

Statistical analyses were performed using R (version 3.4.1; *https://www.R-project.org/*). All data are presented as mean ± standard deviation or median and interquartile range as appropriate. VS and MCS patients’ characteristics were compared using the t-test for normally distributed or the Mann-Whitney U-test for non-normally distributed data and the χ^2^ test for categorical variables. Because patient’s death could prevent the observation of the event “emergence”, variables associated with the outcome were evaluated using survival competing risk models, in particular, cumulative incidence function for univariate analysis [27, 28] and Fine-Gray hazard model for multivariate analysis [29]. To fulfil the assumption of the survival models about non-informative censoring, since the duration of rehabilitation program was strongly influenced by the patient condition at admission, the study follow-up was limited at the first 8 weeks after admission. To identify subgroups of patients with different levels of likelihood of emergence from DoC, a survival tree approach was used as described in Lofaro et al. [30]. Discrimination model performance was measured using the concordance index (C index), a measure equivalent to the area under the receiver operating characteristic curve in logistic regression [31]. Predictive models were trained on the subset of patients admitted till December 2014 (training set) and C index was calculated on the remaining patients admitted between 2015 and 2017 (test set). For all tests a *p* < 0.05 value was considered to be statistically significant.

## Results

### Clinical data

**Fig. 2:**
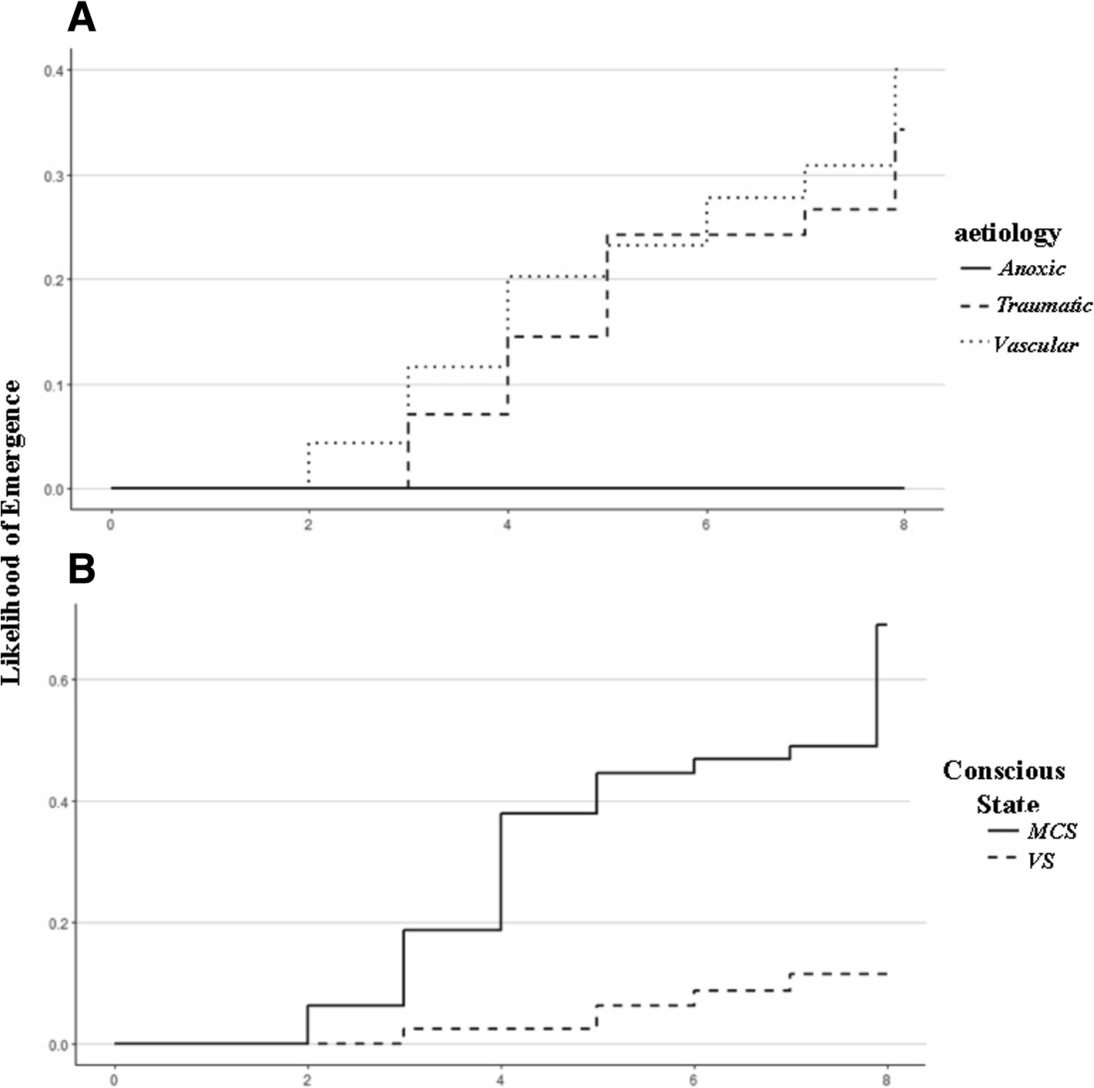
Likelihood of emergence measured with Coma Recovery Scale-Revised (CRS-R) by (**a**) DoC aetiology and (**b**) conscious state at admission

A total of 180 patients were enrolled in the study. Mean follow-up was 6.8 ± 2.0 weeks. Patient and injury characteristics are described in Table 1. 14% of DoC patients showed paroxysmal sympathetic hyperactivity signs, which were treated with beta-blockers, baclofen, clonidine, gabapentin which, however, did not affect the responsiveness of patients. Other neurological complications (eg, hydrocephalus, infections, epileptic attacks) could be detected and were immediately treated appropriately to reduce the risk of further disability. At admission the aetiology of DoC patients, both in VS and MCS, were predominately vascular, followed by traumatic injuries. The vascular cohort consisted of 13% ischemic stroke; 27% poor grade of subarachnoid hemorrhage; 52% Intraparenchymal hemorrhage; 8% subdural hemorrhage. VS and MCS groups resulted matched for demographical and clinical (i.e., aetiology and route of feeding) variables. Eight weeks after admission, 5 (2,7%) patients were dead, 64 (35.5%) had a full recovery of consciousness and 111 (61.7%) remained in VS or MCS. Cumulative incidence of emergence at 4 and 8 weeks after rehabilitation admission by DoC aetiology was 20.5 and 41.6% for vascular and 16.7 and 31.8% for traumatic aetiology, while none of the anoxic patients recovered (*p* = 0.0007; Fig. 2a). According to consciousness state cumulative incidence for VS and MCS patients was 10.6 and 39% after 4 weeks and 21.3 and 68.6% after 8 weeks (*p* < 0.0001; Fig. 2b).

**Table 1:** Clinical characteristics of the study cohort and stratified by conscious state at admission

	Cohort *n* = 180	VS *n* = 123	MCS *n* = 57	p*
Age (years)	51.1 ± 17.3	50.1 ± 16.4	53.1 ± 19.2	0.279
Male (%)	120 (66.7)	84 (68.3)	36 (63.2)	0.610
Etiology (%)				0.079
Traumatic	54 (30.0)	37 (30.1)	17 (29.8)	
Anoxic	24 (13.3)	21 (17.1)	3 (5.3)	
Vascular	102 (56.7)	65 (52.8)	37 (64.9)	
Days in IRU (%)				0.974
< 31	71 (39.4)	49 (39.8)	22 (38.6)	
31–59	83 (46.1)	56 (45.5)	27 (47.4)	
60–89	26 (14.4)	18 (14.6)	8 (14.0)	
Route of Feeding (%)				0.031
PF	7 (3.9)	5 (4.1)	2 (3.5)	
NGT	118 (65.6)	73 (59.3)	45 (78.9)	
PEG	55 (30.6)	45 (36.6)	10 (17.5)	
Tracheostomy (%)	164 (91.1)	117 (95.1)	47 (82.5)	0.013
CRS	6.0 (4.0–9.0)	5.00 (3.0–6.0)	11.0 (9.0–12.0)	< 0.001
Follow-up (weeks)	6.8 ± 2.0	7.4 ± 1.4	5.6 ± 2.4	< 0.001

*p-value referred to statistical comparison between VS Vs *MCS groups*

VS*: Vegetate State; MCS: Minimal Conscious State; IRU: Intensive Rehabilitation Unit; PF: parenteral feeding; PEG: percutaneous endoscopic gastrostomy; NGT: nasogastric tubes*

At univariate analysis, the diagnosis, the CRS-r scores together with aetiology of disease were significantly associated with emergence. Two multivariate survival models were built one with VS/MCS condition and the other with CRS values and both models showed good prediction performance (C-index 0.708 and 0.699 respectively; Table 2).

**Table 2:** Univariate and multivariate Fine-Gray models for the event emergence from altered consciousness state

	Univariate		Multivariate ^†^	
	HR (95% CI)	C Index	HR (95% CI)	C Index
Age (years)	1.01 (0.99–1.03)	0.313	1.00 (0.98–1.02)	
Male (vs female)	0.76 (0.41–1.42)	0.585	0.71 (0.36–1.38)	
MCS (vs VS)	9.36 (4.45–19.69)*	0.613	9.68 (4.46–21.01)*	0.708
CRS	1.36 (1.25–1.48)*	0.668	1.38 (1.26–1.52)*	0.699
Aetiology		0.554		
Anoxic (vs. Traumatic)	- ^§^		–	
Vascular (vs. Traumatic)	1.25 (0.66–2.38)		1.00 (0.46–2.18)	
IRU days		0.588		
31–59 (vs < 31)	0.82 (0.43–1.56)		0.56 (0.28–1.17)	
60–89 (vs < 31)	0.43 (0.15–1.27)		0.36 (0.12–1.11)	
Tree Subgroups		0.604		
Subgroup B (vs A)	6.27 (0.61–842.86)			
Subgroup C (vs A)	10.33 (1.22–1346.99)*			
Subgroup D (vs A)	33.67 (4.57–4294.73)*			
Subgroup E (vs A)	81.77 (11.01–10,442.44)*			

*† Both multivariate models have been developed with covariates Age, Sex, Aetiology, ICU days and, alternatively, MCS/*VS *state or CRS value at admission*

*: p < 0.05; § p < 0.05 for the k-sample test comparing the subdistribution for the event emergence

*HR: Hazard-Ratio; IRU: Intensive Rehabilitation Unit; MCS: minimally* consciousness state; VS*: Vegetative State; CRS: Coma-Recovery Scale*

**Figure 3:**
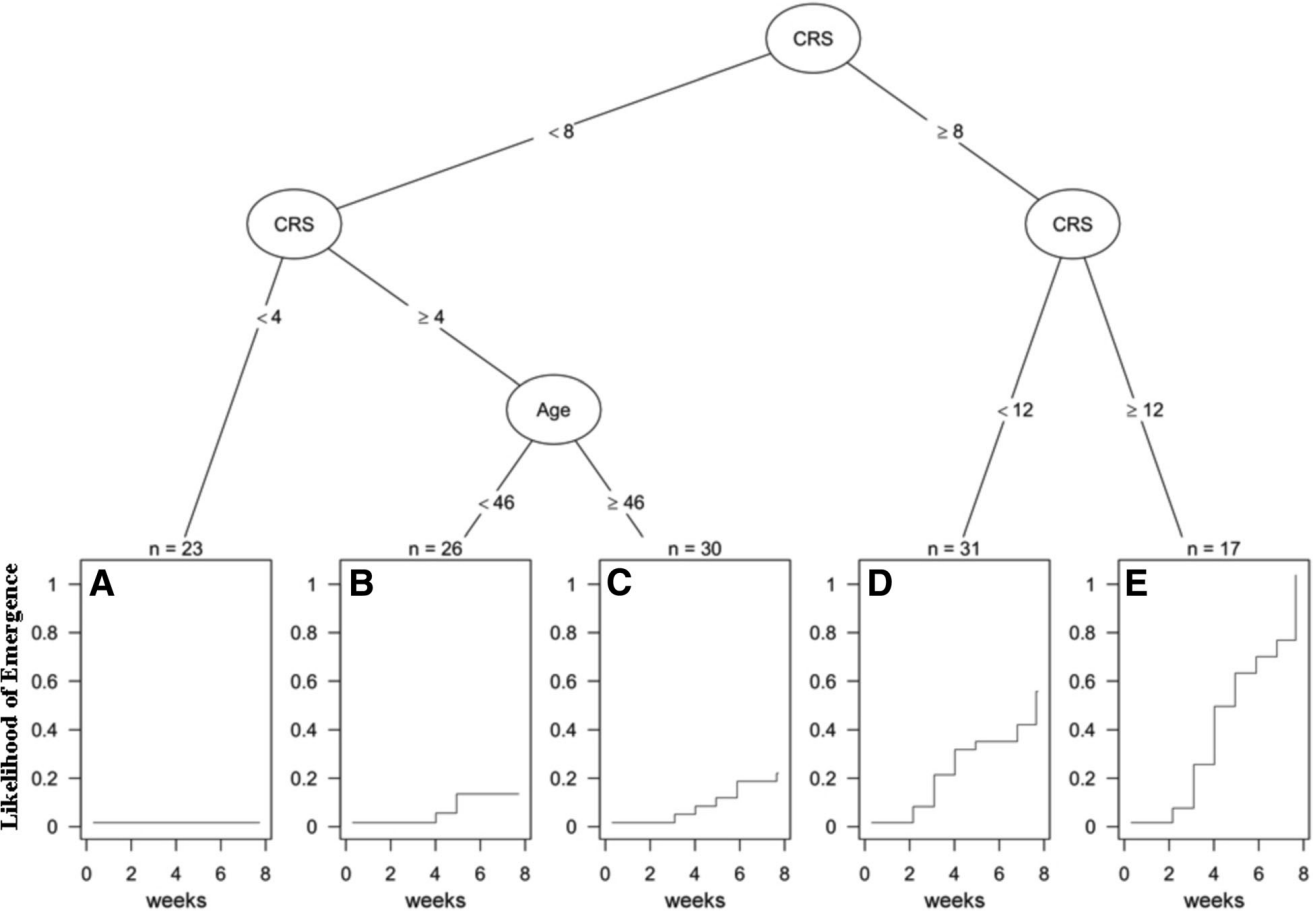
Survival tree for the event “Emergence” from DoC. Terminal panels show the cumulative incidence of Emergence of patients part of the subgroups defined by the conditions on the edges versus all other patients in the cohort

To identify subgroup of patients with different incidence of emergence, a survival tree approach was used. The algorithm identified five subgroups: those with CRS at admission < 4 (A; cumulative incidence at 8 weeks = 0%), patients with CRS 4–7 and age < 46 (B; 11.5%, *p* = 0.239 vs. subgroup A) or ≥ 46 (C; 20%; *p* = 0.043), patients with CRS between 8 and 11 (D; 53%; *p* = 0.017) and those with a CRS ≥ 12 (E; 100%; *p* = 0.003). This stratification of patients showed good discrimination ability (C-index 0.604), but lower than multivariate survival models (Fig. 3).

## Discussion

The current literature on the outcome predictors of the emergence from DoC is in its moderate infancy and nowadays reliable markers have not been fully identified [9–21]. After brain injury events, the early stages are crucial to determine the severity of the disease and to help clinicians and patients’ family in decision-making processes. Indeed, after discharge from IRUs, a better understanding of clinical evolution is mandatory for guiding decisions about pharmacological treatment and rehabilitation planning [9]. The emergence from MCS is represented by return of functional communication or by the ability to use objects functionally (Giacino et al. [4]). Recovery of interactive communication may occur through yes–no responses to questions concerning personal orientation, writing or verbalization. However, the likelihood of emergence from DoC is highly variable depending on several factors, such as: aetiology, age at onset, duration of DoC or clinical complications. In our Italian sample, the detected amount of full recovery of consciousness (35.5%) was in line with previous studies [17, 18, 31] although a final assessment requires a larger temporal period after injury.

Generally, the clinical evolution in this kind of patients is complicated by several factors (i.e., epileptic seizure, infection, thrombosis, paroxysmal sympathetic hyperactivity), which strongly reduces the likelihood of emergence. For instance, dysautonomia crises are common [32] and can be difficult to treat. However, in the last two decades, some clinical variables have been recognized as predictive of a favourable functional improvement. As elegantly summarized by Estraneo and Trojano [31], the usefulness of neurophysiological markers as extracted by EMG, EEG or fMRI methods have been widely recognized [33, 34], although they are rarely used in traditional IRUs. Considering only clinical variables, it has widely been recognized that diagnosis of VS, anoxic aetiology, older age and large temporal interval from the event are the most negative prognostic factors in DoC patients [13, 35, 36]. Overall our data are in part in agreement with all previous studies assessing predictive factors of conscious awareness in short-term period [17–19].

However, our most important finding was that the CRS-r score is the best predictor of clinical improvement as revealed by multivariate survival tree statistical approach. In particular, we found that scores ≥12 at admission are highly predictive of emergence in DoC patients after discharge. This finding enlarges previous evidence provided by Estraneo et al., [37], who only investigated DoC with anoxic aetiology, demonstrating that CRS-r scores higher than 6, was the best predictor of recovery of consciousness. On the other hand, Bodien et al., [20] demonstrated that a total CRS-r scores ≥10 should be considered as a marker of conscious awareness either for diagnosis of MCS or for assessing the emergence from MCS.

Otherwise, with respect to previous literature, we did not confirm that length of stay in the IRU impacts functional outcome [38]. In this work, the authors evaluated the rehabilitation outcome in 63 DoC patients. They found that younger age, shorter stay in the IRU, and MCS diagnosis at admission were found to be significant predictors for higher functional motor improvement at discharge. However, with respect to our study, they only investigated DoC patients with aneurysmal subarachnoid haemorrhagic aetiology, which basically present a different clinical evolution with respect to traumatic patients.

Our study has some limitations that deserve to be discussed. The main limitation of this study is that the outcomes at 2 months cannot be considered as definitive. Although the detected predictors are similar to those reported in long-term longitudinal studies [9–21], we are aware that this study needs further evaluation before translating to clinical practice. Again, the lack of a deeper evaluation of medical complications might have influenced our data. However, it is important to bear in mind that endocrine, metabolic or other neurological complications (i.e seizures) might be reported in the later phases of the disease [12]. Finally, the employment of a retrospective study design is more subject to confounding. For instance, other risk factors could be present that were not measured, such as EEG evaluations. Indeed, it has been demonstrated that EEG coherence might have a diagnostic value in the prognosis of recovery from VS [39], its inclusion might have been improved the strength of our predictive model.

## Conclusions

We demonstrated in a large sample that 35% of DoC patients achieve a full functional improvement by the end of inpatient rehabilitation and that this clinical evolution at discharge was predicted by specific clinical factors at 8 weeks. In particular, our study highlights the importance of CRS-r in the clinical management of DoC, demonstrating its positive prognostic value in post-comatose brain-injured patients. Further evaluations are currently being put together in order to determine how these initial outcomes can change in relationship with DoC evolution.

## Abbreviations

CRS-r: Coma Recovery Scale-Revised
DoC: disorders of consciousness
IRU: intensive rehabilitation unit
MCS: minimal conscious state
VS: vegetative state
